# Standardizing operational vector sampling techniques for measuring malaria transmission intensity: evaluation of six mosquito collection methods in western Kenya

**DOI:** 10.1186/1475-2875-12-143

**Published:** 2013-04-30

**Authors:** Jacklyn Wong, Nabie Bayoh, George Olang, Gerry F Killeen, Mary J Hamel, John M Vulule, John E Gimnig

**Affiliations:** 1 Centers for Disease Control and Prevention, Division of Parasitic Diseases and Malaria, Atlanta, GA, USA; 2 Kenya Medical Research Institute, Centre for Global Health Research, Kisumu, Kenya; 3 Liverpool School of Tropical Medicine, Vector Group, Liverpool, UK; 4 Ifakara Health Institute, Environmental Sciences Thematic Group, Ifakara, United Republic of Tanzania

**Keywords:** *Anopheles gambiae*, *Anopheles arabiensis*, *Anopheles funestus*, Mosquito sampling, Human biting rate, Human landing catch, CDC light trap, Ifakara tent trap, Window exit trap

## Abstract

**Background:**

Operational vector sampling methods lack standardization, making quantitative comparisons of malaria transmission across different settings difficult. Human landing catch (HLC) is considered the research gold standard for measuring human-mosquito contact, but is unsuitable for large-scale sampling. This study assessed mosquito catch rates of CDC light trap (CDC-LT), Ifakara tent trap (ITT), window exit trap (WET), pot resting trap (PRT), and box resting trap (BRT) relative to HLC in western Kenya to 1) identify appropriate methods for operational sampling in this region, and 2) contribute to a larger, overarching project comparing standardized evaluations of vector trapping methods across multiple countries.

**Methods:**

Mosquitoes were collected from June to July 2009 in four districts: Rarieda, Kisumu West, Nyando, and Rachuonyo. In each district, all trapping methods were rotated 10 times through three houses in a 3 × 3 Latin Square design. Anophelines were identified by morphology and females classified as fed or non-fed. *Anopheles gambiae s.l.* were further identified as *Anopheles gambiae s.s.* or *Anopheles arabiensis* by PCR. Relative catch rates were estimated by negative binomial regression.

**Results:**

When data were pooled across all four districts, catch rates (relative to HLC indoor) for *An. gambiae s.l* (95.6% *An. arabiensis*, 4.4% *An. gambiae s.s*) were high for HLC outdoor (RR = 1.01), CDC-LT (RR = 1.18), and ITT (RR = 1.39); moderate for WET (RR = 0.52) and PRT outdoor (RR = 0.32); and low for all remaining types of resting traps (PRT indoor, BRT indoor, and BRT outdoor; RR < 0.08 for all). For *Anopheles funestus,* relative catch rates were high for ITT (RR = 1.21); moderate for HLC outdoor (RR = 0.47), CDC-LT (RR = 0.69), and WET (RR = 0.49); and low for all resting traps (RR < 0.02 for all). At finer geographic scales, however, efficacy of each trap type varied from district to district.

**Conclusions:**

ITT, CDC-LT, and WET appear to be effective methods for large-scale vector sampling in western Kenya. Ultimately, choice of collection method for operational surveillance should be driven by trap efficacy and scalability, rather than fine-scale precision with respect to HLC. When compared with recent, similar trap evaluations in Tanzania and Zambia, these data suggest that traps which actively lure host-seeking females will be most useful for surveillance in the face of declining vector densities.

## Background

The entomological inoculation rate (EIR), expressed as the number of infectious mosquito bites per person per unit time, is a direct measure of malaria transmission intensity and, hence, an important metric for malaria surveillance and control programme evaluation [1,2]. Calculating EIR requires trapping host-seeking *Anopheles* vectors to determine the human biting rate (HBR) and prevalence of sporozoite infection [1,3,4]. Methods for operational mosquito sampling currently lack standardization, however, making it difficult to quantitatively compare EIRs between different locations or even within one location over time [2]. The gold standard in research studies for estimating mosquito-human contact has traditionally been the human landing catch (HLC), which can be employed either indoors or outdoors to capture mosquitoes as they land to feed on a human host [5,6]. Unfortunately, HLC is a labour-intensive procedure requiring highly trained collectors and extensive supervision, and results can be biased due to differences in the skill of collectors or their attractiveness to mosquitoes [2,7,8]. Furthermore, use of HLC has declined in recent years due to ethical concerns about potential exposure of collectors to mosquito-borne pathogens [5,9]. For all these reasons, HLC is unsustainable for large-scale operational sampling of malaria vectors.

Several exposure-free trapping methods have been evaluated as substitutes for HLC, but their reliability for estimating HBR and sporozoite prevalence have varied widely depending on characteristics of the study location or vector population [9,10]. Centers for Disease Control and Prevention miniature light traps (CDC-LT) are commonly used and are most efficient when hung next to a human host that is protected under a bed net [11,12]. Several studies have demonstrated close correlation between the numbers of *Anopheles* mosquitoes caught by CDC-LT compared to HLC [10-15]. Others researchers, however, have reported low catch rates [16,17] or inconsistent results using CDC-LT [18]. Also of concern, CDC-LTs have been found to catch higher numbers of *Plasmodium*-infected females compared to HLC [16,19], potentially resulting in overestimation of EIR.

Window exit traps (WETs) and resting traps are easy to deploy and do not require a dedicated collector to act as human bait [6,20]. Neither of these trap types, however, specifically target host-seeking females. WETs capture endophagic or endophilic vectors as they leave the house after feeding and/or resting. Resting traps are particularly useful for studying mosquito host choice behaviour because they collect large proportions of fed females immediately after a blood meal [6]. The efficacy of WETs and resting traps are strongly affected by environmental variables, such as the number of alternative exits or resting sites available for mosquitoes [6,17,21]. The C-design of the Ifakara tent trap (ITT) was recently developed as an alternative exposure-free tool to collect host-seeking malaria vectors [22]. While this tent trap has demonstrated high capture rates for *Anopheles* in both rural and urban study sites in Tanzania [22,23], its efficacy is known to vary depending on local vector density [24] and only one evaluation outside of Tanzania has been conducted in neighbouring Zambia [15].

The aim of the present study was to compare mosquito catch rates from CDC-LT, ITT, WET, pot resting trap (PRT), and box resting trap (BRT) against HLC in four different districts in western Kenya to identify the most useful tools for operational surveillance in this region of declining vector density. By employing a study design similar to recent trap evaluations in Tanzania [17] and Zambia [15], the secondary goal was to provide information enabling comparisons of trap efficacy between countries, an important step toward standardizing vector collection techniques across multiple sites.

## Methods

### Study area

**Figure 1:**
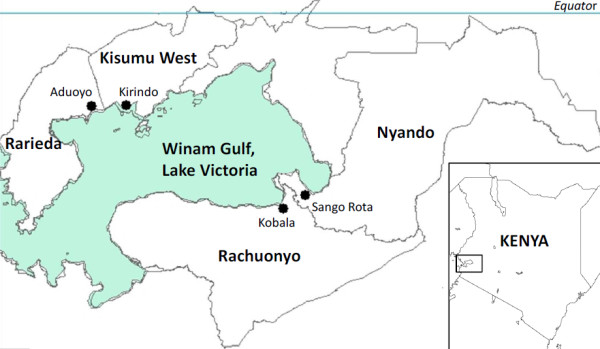
**Map of western Kenya. **The four study districts are shown in bold. Within each district, the village in which mosquitoes were collected is marked with a dot and labelled.

This study was conducted in four districts in rural western Kenya: Rarieda, Kisumu West, Nyando, and Rachuonyo (Figure 1). Rarieda, located on the north side of the Winam Gulf of Lake Victoria, is an area of intense year-round malaria transmission. Several epidemiological, entomological and immunological studies of malaria have been conducted in this region [25-29]. EIRs estimated during the early 1990s ranged from 60 to 300 infectious bites per person per year [30,31]. From 1997–1999, residents of Rarieda District (within the Asembo jurisdiction) were enrolled in a community-wide study to determine the efficacy of insecticide-treated bed nets (ITNs) for reducing malaria morbidity and mortality [26]. High ITN coverage (about 90%) was achieved in the study area and maintained programmatically for years after the study [32]. In Rarieda district, EIR estimates have ranged from five to 15 infectious bites per person per year since 2002 despite nearly universal coverage with bed nets (J Gimnig, unpublished). Annual malaria parasite prevalence in children under five years of age reached a low of 25% in 2008, but was over 40% in 2009 and 2012 (M Hamel, unpublished data). Kisumu West borders Rarieda and entomological monitoring suggests that malaria transmission is similar to that of Rarieda.

Nyando and Rachuonyo are on the eastern and southern sides of the gulf, respectively, where low to moderate malaria transmission is observed. EIRs have not been estimated for these areas, but routine entomological surveys indicate that average mosquito densities are consistently 75% lower than in Rarieda. Malaria parasite prevalence in these districts is also lower than in Rarieda; prevalence across all age groups was estimated to be less than 10% in May 2008. In August 2009, parasite prevalence remained under 10% in Rachuonyo District following two rounds of indoor residual spraying (IRS) with pyrethroid insecticides, but rose to approximately 16% in neighbouring Nyando District where IRS was not conducted (J Gimnig, unpublished data).

Previously, bed net ownership and use was highest in Rarieda due to the ITN study conducted in that district. However, after a mass campaign in 2006 and several years of targeted subsidies to pregnant women and children, household ownership of ITNs in most of western Kenya approached that of Rarieda [33]. Malaria vectors in this region include *Anopheles gambiae s.s.*, *Anopheles arabiensis* and *Anopheles funestus*, with *An. gambiae s.s.* predominant through the late 1990s [30,33]. Following widespread distribution of ITNs, however, populations of *An. gambiae s.s.* and *An. funestus* declined dramatically, leaving *An. arabiensis* as the most abundant vector species at present [33]. Two rounds of IRS with pyrethroids were carried out in all houses in Rachuonyo District in July/August 2008 and April/May 2009. No IRS was conducted in Rarieda, Kisumu West, or Nyando prior to this study (J Gimnig, unpublished data). Mosquito collections were conducted from June to July 2009, corresponding to the end of the long rainy season.

### Experimental design

**Figure 2:**
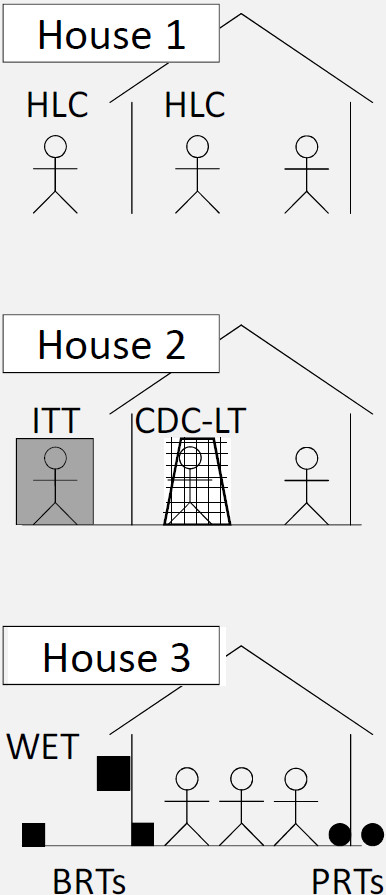
**Schematic diagram of experimental set up within one district. **A block of three locally representative houses was used to evaluate the following combinations of trapping methods: (1) HLC indoor and HLC outdoor, (2) CDC-LT indoor (placed beside an occupied bed net) and ITT outdoor, and (3) WET combined with two PRTs (one indoor and one outdoor) and two BRTs (one indoor and one outdoor). Trapping methods were rotated through all houses according to a 3 × 3 Latin Square design. Aside from the person acting as bait for the CDC-LT, all residents were free to choose whether or not to sleep under their pre-existing bed nets.

To facilitate comparison with recent trap evaluations in Tanzania [17] and Zambia [15], a similar study design was employed. Within each of the four study districts, a block of three locally representative houses was used to evaluate vector trapping methods over a 30-day period. Study houses were selected in areas that either provided abundant mosquitoes in the past or were in close proximity to breeding sites. The following paired combinations of trapping methods were used during the study: (1) HLC indoor and HLC outdoor; (2) CDC-LT indoor (hung beside an occupied bed net) and ITT outdoor (Elastic Products Manufacturing Co, Dar es Salaam, United Republic of Tanzania); and (3) WET combined with two PRTs (one indoor and one outdoor) and two BRTs (one indoor and one outdoor) (Figure 2). These collection techniques have been described in detail previously [15,17,20]. The ITT consisted of a canvas tent fitted with six funnel-shaped entrances for mosquitoes. To prevent sleepers from being bitten by mosquitoes, the ITT C-design used in this study was a modification of the original ITT such that each mosquito entrance led to one of two screened compartments [22]. PRTs were locally manufactured clay pots that have previously been evaluated for sampling *Anopheles* in western Kenya [20]. Trap methods were rotated 10 times through the three houses every three nights in a 3 × 3 Latin Square design.

In each district, five adult male volunteers were recruited to carry out mosquito collections. HLCs were conducted from 18:00 to 06:00 each night, with 45 min of collection and a 15 min break per hour. The same four men conducted HLCs every night, with two men (one indoor and one outdoor) collecting during the first shift (18:00 to 00:00) and two men (one indoor and one outdoor) collecting during the second shift (0:00 to 06:00). The four HLC collectors were rotated between positions (indoor *vs* outdoor) and shifts (first *vs* second) to account for potential differences in their attractiveness to host-seeking mosquitoes. The fifth volunteer carried out collections using the ITT each night.

To utilize representative natural conditions as much as possible, local houses with their normal occupants were used as sampling locations. In addition, bed nets already present in houses (some insecticide-treated and some untreated) were used in this study and no new nets were provided. Aside from the person acting as bait for the CDC-LT (who was required to use their pre-existing net), residents were free to choose whether or not to sleep under their bed nets each night. Bed net usage and insecticide treatment was recorded at the end of the study. Throughout the entire study, each house was occupied by at least three adults, of whom at least two were adult males. All volunteers, notably those conducting HLCs, were supervised through random spot checks throughout the study.

For HLCs, each hourly catch from each volunteer was placed in a separate cup. Mosquitoes were aspirated from PRTs and BRTs, and collected from the CDC-LT, ITT, and WET, between 06:00 and 07:00 following each sampling night.

### Mosquito processing

Captured *Anopheles* mosquitoes were identified to species morphologically [34,35]. For *Anopheles gambiae s.l*, individuals were identified to sibling species level by PCR [36]. All female anophelines were classified by abdominal status (fed, unfed, gravid, or half-gravid). The prevalence of *Plasmodium falciparum* sporozoites in collected anophelines was determined by sandwich ELISA [37]. *Culex* mosquitoes were classified as male or female and then discarded.

### Data analysis

Data were entered into a Microsoft Access database (Microsoft, Redmond, WA, USA) and analysed using SAS version 9.1 (SAS Institute, Cary, NC, USA). Mosquito catch rates relative to HLC indoor were calculated by fitting negative binomial regression models using PROC GENMOD. The number of adult females caught in each trap on each night was treated as the outcome variable and separate regression models were fitted for each species. Models employed an autoregressive correlation structure allowing for correlation to decline with increasing time between collections. For all models, house was used as the repeated subject. Trapping method and district were included as predictor variables. If any trap method by district interactions were significant (p < 0.05), relative catch rates were reported separately for each district. Characteristics of household bed net usage, including type of bed net (insecticide-treated or untreated) and whether all residents slept under a bed net (yes or no), were also examined for influence on trap efficacy.

To test for an effect of trapping method on the proportion of captured females that had recently fed, logistic regression models were fitted separately to the *An. gambiae s.l* and *An. funestus* catch data. Abdominal status of females was coded as either fed or non-fed (unfed, gravid, and half-gravid). Trapping method and district were included as predictor variables and models were fitted using an autoregressive correlation structure with house as the repeated subject to calculate odds ratios for catching fed females.

### Ethical clearance

The study protocol was approved by the Kenya Medical Research Institute Ethical Review Committee (#1399). The Centers for Disease Control and Prevention reviewed the protocol and determined that it did not constitute human subjects research (#990153). All collectors consented to participating in the study and were provided mefloquine on a weekly basis as malaria prophylaxis.

## Results

### Mosquito populations in western Kenya

Due to unavailability of PRTs and BRTs in some districts at the beginning of the study, several sampling nights lacked the full complement of trap types. Those sampling nights were excluded, resulting in analysis of mosquito collections from 106 nights with complete trap data (24 nights from Rarieda, 23 nights from Kisumu West, 30 nights from Nyando, and 29 nights from Rachuonyo).

Of the 15,706 female mosquitoes collected, most were *Culex* spp. (92.5% of females; n = 14,525). *Anopheles* species captured included *An. gambiae s.l* (6.4% of females; n = 1,012), *An. funestus* (1.1% of females; n = 165), and *Anopheles coustani* (<0.1% of females; n = 4). While *An. gambiae s.l* and *An. funestus* were prevalent across all four districts, *An. coustani* was found only in Nyando (n = 2) and Rachuonyo (n = 2). Due to the small number of *An. coustani* captured, statistical analyses were not conducted for this species. Out of 339 successfully amplified *An. gambiae s.l.* females, 95.6% were *An. arabiensis* (n = 324) and 4.4% were *An. gambiae s.s* (n = 15). Thus, the subsequent results for *An. gambiae s.l* in this region are generalizable to *An. arabiensis*.

### Relative catch rates for traps compared to HLC indoor

The numbers of females caught by each trapping method are summarized in Table 1. All 12 households in the study reported owning at least one bed net. Whether or not all household residents slept underneath a net had no impact on trap efficacy (*An. gambiae s.l*, p = 0.487; *An. funestus*, p = 0.275, *Culex* spp., p = 0.543) and this factor was excluded from the models. Because only two households (both in Rachuonyo) reported using nets that were insecticide-treated (either long-lasting insecticide-treated nets (LLINs) less than three years old or nets treated within the last 12 months), potential impacts of treated *vs* non-treated nets on trap efficacy could not be evaluated. The assessment of net treatment status in this study, however, may have underestimated the true number of treated nets. A mass LLIN distribution campaign was conducted during the second half of 2006, which significantly increased ITN ownership throughout all of Kenya [38]. LLINs from this campaign had just exceeded the three-year mark by the end of the trap evaluation, and thus, were counted as untreated. Furthermore, while large numbers of untreated nets had been distributed by KEMRI\CDC in the Asembo Bay area of Rarieda, these nets were treated with the KO-Tab 123 (a long-lasting insecticide formulation) [39] in early 2007. Therefore, it is likely that many of the bed nets used during this study still exhibited insecticidal activity, but were reported as untreated to err on the side of caution.

**Table 1:** Numbers of female mosquitoes caught using different trapping methods

**Trapping method**	**Total catch**	**Mean catch per night (95% CI)**	**Relative capture rate (95% CI)**	**p-value**
*Anopheles gambiae s.l.*				
HLC indoor	208	1.96 (1.39, 2.54)	1.00^*^	NA
HLC outdoor	180	1.70 (1.13, 2.27)	1.01 (0.76, 1.34)	0.947
CDC light trap	225	2.12 (1.36, 2.89)	1.18 (0.55, 2.54)	0.666
Ifakara Tent Trap	213	2.01 (1.55, 2.47)	1.39 (0.88, 2.19)	0.153
Window exit trap	117	1.10 (0.66, 1.54)	**0.52 (0.30, 0.91)**	**0.022**
Pot resting trap indoor	8	0.08 (0.01, 0.14)	**0.05 (0.02, 0.11)**	**<0.001**
Pot resting trap outdoor	48	0.45 (0.26, 0.64)	**0.32 (0.12, 0.87)**	**0.025**
Box resting trap indoor	0	0.00^#^	0.00^#^	NA
Box resting trap outdoor	13	0.12 (0.05, 0.19)	**0.08 (0.04, 0.14)**	**<0.001**
*Anopheles funestus*				
HLC indoor	43	0.41 (0.20, 0.61)	1.00^*^	NA
HLC outdoor	20	0.19 (0.05, 0.33)	**0.47 (0.32, 0.70)**	**<0.001**
CDC light trap	29	0.27 (0.12, 0.43)	**0.69 (0.49, 0.98)**	**0.04**
Ifakara Tent Trap	52	0.49 (0.27, 0.71)	1.21 (0.88, 1.68)	0.245
Window exit trap	20	0.19 (0.08, 0.30)	**0.49 (0.36, 0.65)**	**<0.001**
Pot resting trap indoor	0	0.00^#^	0.00^#^	NA
Pot resting trap outdoor	1	0.01 (0.00, 0.03)	**0.02 (0.00, 0.24)**	**0.001**
Box resting trap indoor	0	0.00^#^	0.00^#^	NA
Box resting trap outdoor	0	0.00^#^	0.00^#^	NA
*Culex *spp*.*				
HLC indoor	4915	46.37 (36.96, 55.78)	1.00^*^	NA
HLC outdoor	6566	61.94 (51.78, 72.10)	**1.49 (1.10, 2.00)**	**0.009**
CDC light trap	1122	10.58 (6.82, 14.35)	**0.21 (0.15, 0.31)**	**<0.001**
Ifakara Tent Trap	1406	13.26 (7.69, 18.84)	**0.38 (0.20, 0.72)**	**0.003**
Window exit trap	271	2.56 (1.35, 3.77)	**0.07 (0.03, 0.13)**	**<0.001**
Pot resting trap indoor	81	0.76 (0.48, 1.05)	**0.02 (0.01, 0.03)**	**<0.001**
Pot resting trap outdoor	80	0.75 (0.38, 1.13)	**0.02 (0.01, 0.04)**	**<0.001**
Box resting trap indoor	26	0.25 (0.06, 0.43)	**0.00 (0.00, 0.01)**	**<0.001**
Box resting trap outdoor	58	0.55 (0.14, 0.96)	**0.01 (0.00, 0.02)**	**<0.001**

Data were combined across all four districts (106 nights for each trap type). Catch rates (relative to HLC indoor), 95% confidence interval, and p-value were calculated using negative binomial regression models. Relative capture rates statistically different from HLC indoor (p < 0.05) are indicated in bold.

^* ^Reference collection method.

^# ^Trap types that captured zero females were excluded from the model for that species.

Mosquito collection data were initially pooled across all districts for analysis. HLC outdoor, CDC-LT, and ITT performed well, with no differences found in the numbers of *An. gambiae s.l.* caught compared to the gold standard of HLC indoor. WET and PRT outdoor yielded fewer *An. gambiae s.l.*, but still captured sufficient numbers to be useful for collection*.* The remaining types of resting traps (PRT indoor, BRT indoor, and BRT outdoor) collected very few *An. gambiae s.l*. Between districts, more *An. gambiae s.l.* were captured in Rarieda compared to Kisumu West (RR = 0.44, p = 0.018), Nyando (RR = 0.37, p < 0.001), and Rachuonyo (RR = 0.09, p < 0.001). Several interaction terms between trap method and district were statistically significant, indicating that trap efficacy for capturing *An. gambiae s.l.* (relative to HLC indoor) varied by district. Table 2 shows the relative catch rate of each trapping method stratified by district.

**Table 2:** **Relative rates for capturing female *Anopheles gambiae s.l. *stratified by district**

**Trapping method**	**Rarieda**	**Kisumu West**	**Nyando**	**Rachuonyo**
HLC indoor	1.00^*^	1.00^*^	1.00^*^	1.00^*^
HLC outdoor	**0.77 (0.63, 0.95)**^a^	0.60 (0.32, 1.11)^a^	1.19 (0.94, 1.52)^b^	**2.57 (1.62, 4.06)**^c^
CDC light trap	**0.76 (0.61, 0.96)**^a^	**2.74 (1.04, 7.23)**^b^	0.54 (0.20, 1.42)^a^	0.36 (0.06, 2.14)^a,b^
Ifakara Tent Trap	**0.48 (0.38, 0.62)**^a^	1.94 (0.99, 3.79)^b^	**1.65 (1.16, 2.35)**^b^	**3.08 (1.02, 9.35)**^b^
Window exit trap	0.72 (0.41, 1.28)^a,b^	**0.18 (0.05, 0.64)**^a^	**0.37 (0.25, 0.56)**^a^	**0.72 (0.64, 0.81)**^b^
Pot resting trap indoor	**0.02 (0.00, 0.07)**^a^	0.00^#^	**0.09 (0.05, 0.16)**^b^	0.53 (0.28, 1.01)^c^
Pot resting trap outdoor	**0.04 (0.01, 0.10)**^a^	**0.02 (0.00, 0.26)**^a^	1.05 (0.51, 2.17)^b^	0.92 (0.20, 4.35)^b^
Box resting trap indoor	0.00^#^	0.00^#^	0.00^#^	0.00^#^
Box resting trap outdoor	**0.05 (0.04, 0.06)**^a^	0.00^#^	**0.11 (0.10, 0.13)**^b^	0.59 (0.18, 1.96)^c^

Catch rate (relative to HLC indoor) and 95% confidence interval were calculated using negative binomial regression models. Within each district (column), trap catch rates statistically different from HLC indoor (p < 0.05) are indicated in bold. Across each trap type (row), different letters denote relative capture rates varying significantly (p < 0.05) between districts. All houses in Rachuonyo District were covered by two rounds of indoor residual spraying (IRS) using pyrethroid insecticides in July/August 2008 and April/May 2009. IRS was not conducted in Rarieda, Kisumu West, or Nyando districts.

^* ^Reference collection method within each district.

^# ^Trap types that captured zero females were excluded from the model for that district.

*Anopheles funestus* were far more abundant in Rarieda compared to Kisumu West (RR = 0.03, p < 0.001), Nyando (RR = 0.02, p < 0.001), or Rachuonyo (RR = 0.03, p < 0.001), so patterns in trap efficacy primarily reflect those in Rarieda. ITT was the only trap method for which the number of *An. funestus* captured was not significantly different from HLC indoor (Table 1). CDC-LT, WET, and HLC outdoor caught significantly fewer *An. funestus*. Almost no *An. funestus* were caught in pot or box resting traps, regardless of location. Due to low catch rates in three of the four districts, differences in trap efficacy between the four districts could not be evaluated.

For *Culex* spp, HLC outdoor captured more adult females than the gold standard of HLC indoor (Table 1). CDC-LT and ITT caught fewer *Culex* spp compared to HLC indoor, but still yielded appreciable numbers. WET and all resting traps performed poorly. Compared to Rarieda, the number of *Culex* captured was not significantly different in Kisumu West (RR = 1.55, p = 0.059) and Rachuonyo (RR = 0.59, p = 0.088), but was significantly higher in Nyando (RR = 2.99, p < 0.001). The efficacy of some traps for capturing *Culex* spp (relative to HLC indoor) varied by district. District-stratified catch rates for each trapping method are presented in the Additional file 1.

### Sporozoite prevalence rates

During this study, sporozoite-infected *Anopheles* were captured only in Rarieda District. Sporozoite prevalence in this district was 2.0% for *An. gambiae s.l*. (n = 12 infected females) and 2.6% for *An. funestus* (n = 5 infected females). The largest numbers of infected *An. gambiae s.l.* were captured by WET (n = 4, 4.3% infection rate) and CDC-LT (n = 2, 2.0% infection rate). For *An. funestus*, ITT yielded the largest number of infected females (n = 4, 8.2% infection rate). Overall numbers of infected mosquitoes were too sparse to analyze any effect of trap type on sporozoite rates.

### Proportion of fed female anophelines

Among *An. gambiae s.l.*, 20.3% of all captured females had recently blood fed (n = 205). The percentage of recently fed females was 17.0% for *An. funestus* (n = 28). Due to the small number of blood fed *An. funestus* collected, the effect of trapping method on abdominal status was examined only for *An. gambiae s.l.* Almost a fifth of the *An. gambiae s.l.* captured by HLC indoor had recently taken a blood meal (Table 3), a proportion similar to that of HLC outdoor, ITT, and WET. Higher proportions of fed females were caught in resting traps, although the odds ratio for PRT indoor was not statistically different from HLC indoor due to small sample size (Table 3). Compared to HLC indoor, CDC-LT captured a significantly lower proportion of fed *An. gambiae s.l*. Compared to Rarieda, the proportion of fed females was lower in Kisumu West (OR = 0.37, p-value < 0.001), but higher in Nyando (OR = 6.07, p-value < 0.001) and Rachuonyo (OR = 6.46, p-value < 0.001).

**Table 3:** **Effect of trapping method on the proportion of fed *Anopheles gambiae s.l. *captured**

**Trapping method**	**Percentage of females fed**	**OR (95% CI)**	**p-value**
HLC indoor	18.8 (39/208)	1.00^*^	NA
HLC outdoor	17.2 (31/180)	0.71 (0.38, 1.31)	0.274
CDC light trap	5.3 (12/225)	**0.38 (0.16, 0.88)**	**0.024**
Ifakara Tent Trap	17.8 (38/213)	0.73 (0.37, 1.42)	0.355
Window exit trap	27.4 (32/117)	2.09 (0.68, 6.44)	0.198
Pot resting trap indoor	62.5 (5/8)	2.67 (0.18, 40.53)	0.479
Pot resting trap outdoor	81.3 (39/48)	**7.21 (1.65, 31.60)**	**0.009**
Box resting trap indoor	0	NA	NA
Box resting trap outdoor	69.2 (9/13)	**6.54 (2.27, 18.83)**	**<0.001**

Data were combined across all districts. Odds ratios were calculated using logistic regression and are indicated in bold if statistically different from HLC indoor (p < 0.05).

^* ^Reference collection method.

## Discussion

All available mosquito collection methods suffer from some kind of bias or shortcoming [6,40] and there is no consensus regarding which techniques should be applied in which settings [2]. As a result, lack of consistent methodology greatly limits the ability to quantitatively compare EIRs between populations, or even within a population over time [2,40]. Numerous researchers have attempted to calibrate mosquito trapping techniques against the gold standard of HLC [10,18,40-42], but these studies employ varying designs and typically focus on site-specific results rather than facilitating comparisons of trap efficacy between sites [2,15]. With the aim of contributing to a standardized evaluation of mosquito trapping techniques across multiple countries, relative *Anopheles* catch rates from six collection methods were compared using a study design that has also been tested in Tanzania [17] and Zambia [15].

For the purpose of estimating *Anopheles* biting rates in western Kenya, trapping methods specifically targeting host-seeking mosquitoes (i e, CDC-LT and ITT) were the most effective. A high catch rate for CDC-LT is consistent with numerous reports from regions throughout Africa [10-14,24,43], including the recent trap evaluation in Zambia employing a similar Latin Square design [15]. In contrast, the parallel trap evaluation in Tanzania demonstrated low capture rates for CDC-LT in urban Dar es Salaam, an area with many competing light sources [17]. Other researchers have cautioned that light traps may lack sensitivity in areas where mosquito or human populations are particularly sparse [16,18]. Thus, while CDC-LT efficacy is generally expected to be quite high, caution should be used when extrapolating vector biting rates in certain areas that are very well-lit or have unusually low densities of humans or mosquitoes.

Consistent with results from the recent Tanzanian [17] and Zambian [15] evaluations, ITT was highly effective for collecting malaria vectors in western Kenya. A concern was raised, however, that nearly 18% of females caught by ITT had recently fed, a considerably higher percentage than those caught by CDC-LT (which prevents exposure to bites), but a similar percentage to those caught by HLC (which necessitates exposure to mosquito bites). One explanation could be that the ITT C-design did not entirely protect the collector from mosquito bites. In contrast, an ITT evaluation in Tanzania showed that when unfed mosquitoes were released into a semi-field enclosure, all females caught by the ITT C-design remained unfed (i e, females were unable to feed on the collector) [22]. Thus, another explanation could be that already engorged females were trapped by the ITT while attempting to rest or feed again. Further investigation, including analysis of blood meal sources, will be required to address this question definitively.

Although WETs did not specifically target host-seeking females, these traps performed moderately well in western Kenya with pooled relative catch rates of 52% for *An. gambiae s.l* and 49% for *An. funestus*. In contrast, both the Tanzanian and Zambian evaluations reported very low WET catch rates compared with HLCs (1% for *An. gambiae s.l* in Tanzania [17] and 3% for *An. funestus* in Zambia [15]), which the authors attributed to houses having open eaves and numerous points for entry and exit. Houses in western Kenya similarly had open eaves, leaving no clear explanation for the discrepancy between results from the present study and those from Tanzania and Zambia. It is plausible that the houses in western Kenya had narrower gaps between the roof and the walls or fewer windows to serve as alternate exits (only one WET was used per house). By fitting experimental huts in Tanzania with net baffles to allow entry but prevent escape of mosquitoes via eaves, Okumu *et al.*[44] were able to collect similar numbers of mosquitoes in WETs compared to CDC-LTs, suggesting that WETs can be very effective if household exits are limited. Another possibility is that different lighting or wind conditions may affect exit behaviour and WET capture rates. More detailed evaluations of WETs in different types of houses and environmental settings will be necessary for understanding when and where use of WET is reliable.

Resting traps, which primarily capture fed females seeking a secluded place to digest, yielded very few malaria vectors in western Kenya, a pattern also observed in Tanzania [17] and Zambia [15]. A potential explanation is that competing resting sites were plentiful enough indoors (e g, walls, clothing, furniture, etc.) and outdoors (e g, roof, vegetation, etc.) that only small numbers of mosquitoes entered the resting traps. While the absolute numbers of females caught in resting traps were low, the odds ratios for catching fed females in outdoor PRTs and BRTs were six to seven times greater compared to HLC. Thus, resting traps present a trade-off; they are practical for sampling engorged females to study feeding behaviour, host selection, etc. [20], but the numbers caught do not represent quantitative estimates of vector biting density. Even if resting traps accounted for a larger proportion of available resting sites, these traps would likely catch substantially fewer adult *Anopheles* compared to techniques that actively lure host-seeking females [45]. In areas dominated by exophilic vectors, however, one exception might be a novel barrier screen that could be erected between hosts and resting sites (if flight paths can be identified) to collect fed females on one side and host-seeking females on the other side [46].

Wide variation in the relative catch rate of each trapping method was observed between the four districts. Variability in trap performance was likely driven by district-specific differences in ecology, vector species composition, host availability, and/or history of malaria interventions. For example, ITT efficacy is known to decline with increasing vector density [47] and the lowest relative capture rate for ITT was observed in Rarieda, the district with the highest mosquito density. Interestingly, even the relative catch rates for indoor *vs* outdoor HLC differed between districts. Significantly more *An. gambiae s.l* were caught by indoor HLC compared to outdoor HLC in Rarieda, but this pattern was reversed in Rachuonyo, suggesting that outdoor biting was more prevalent in Rachuonyo. This disparity could be due to recent IRS activity (in addition to high bed net coverage) in Rachuonyo selecting for more outdoor feeding compared to Rarieda, where IRS was not conducted. Widespread use of indoor insecticide-based interventions has been correlated with increased exophilic and exophagic behaviour in residual mosquito populations in Tanzania [48], the Solomon Islands [49], and Equatorial Guinea [50]. Detailed analyses of biting location and biting time will be carried out to address whether vector control interventions in western Kenya have promoted changes in mosquito feeding behaviour, as well as the consequences for malaria transmission.

An obvious limitation of this study was that not all mosquito collection methods potentially suitable for operational vector surveillance could be tested. Knockdown pyrethrum spray catches (PSCs) are effective for collecting fed females resting indoors [6] and are commonly conducted throughout Africa [51,52]. Due to issues of insecticide persistence inside houses, however, PSCs were not incorporated into the Latin Square study design. Also promising are traps that employ novel synthetic human odorant blends to attract host-seeking *Anopheles*[53]. By utilizing a standardized a mixture of synthetic odours instead of human bait, such traps may circumvent problems due to differential skill or attractiveness of collectors. The relative attractiveness of odour-baited traps appears to vary with proximity to humans [53], however, and further field testing is required to identify settings appropriate for using these traps.

Despite high overall catch rates in this study for certain trap types (e g, CDC-LT, ITT, and to some extent WET), variability at the district level made it impossible to calculate any simple ‘calibration factor’ to convert trap catches into HLC equivalents consistently across western Kenya. Such variation in trapping efficacy is unavoidable as no method is an exact substitute for HLC [6,10,18,40,42]. Even the catch rate for HLC, the gold standard itself, is not constant; HLC results are influenced by the skill of collectors and their innate attractiveness to mosquitoes [2,7,8]. For the practical purposes of large-scale vector surveillance and operational estimation of EIRs, high overall capture rate and scalability allowing for intensive sampling are likely more important than perfect precision with regard to HLC. Vector populations exhibit marked spatial and temporal heterogeneity, sometimes spanning several orders of magnitude [54-57], such that estimating local population abundance is an inexact science. The challenge of monitoring malaria transmission intensity entomologically is further compounded as vector density and sporozoite infection prevalence decrease in response to successful malaria interventions; mosquito population distribution becomes even more sparse and patchy so that remaining infected mosquitoes are harder to find [54]. This point is well illustrated in western Kenya, where Anopheline populations have declined dramatically since the late 1990’s due to scale-up of ITNs [33]. As a result of sparse mosquito populations, collectors in the present study were unable to capture any infected females in Kisumu West, Nyando, or Rachuonyo (leading to EIR estimates of zero in those districts), despite timing collections to correspond with peak seasonal mosquito density. Malaria transmission was on-going in all four districts, indicating that these study results underestimated the true risk of infection in at least three districts.

Comparison of trap efficacy results (relative to HLC) from western Kenya to those from Tanzania and Zambia highlights the difficulty in estimating calibration factors for even the most widely-used, well-standardized trapping methods, such as the CDC-LT. One interpretation of these findings is that calibration factors must be validated before use in new geographic areas. Variability at very fine spatial and temporal scales, however, suggests that calibration factors may be inherently unreliable. Inter-district variation was observed for nearly all trap types tested in this small region of western Kenya. Furthermore, even a series of three exercises to calibrate CDC-LT against HLC within a single Tanzanian village yielded quite different results over the span of two and a half years [24,58]. Thus, rather than attempting to convert all results to HLC equivalents, vector biologists may be better served by employing consistent trapping methods over time and detailing the specific method they used to obtain EIR values. Concerns have been voiced regarding whether EIRs calculated directly from alternative trap methods are accurate with respect to ‘true’ EIRs from HLCs. To achieve sufficient spatial and temporal resolution, large-scale entomological surveillance programmes require frequent sampling [59] with traps that are inexpensive and easy to use with little or no expert supervision [15,17]. Because non-HLC trap methods afford greatly increased sampling effort, these cost-effective tools may in fact provide better indications of local malaria transmission. Recent evaluation of a routine vector surveillance programme in Tanzania using the ITT revealed that, despite low ITT catch rates per night of trapping compared to HLC, intensive and geographically extensive sampling with ITT was far more accurate for predicting risk of malaria parasite infection than the limited number of quality-controlled HLC collections conducted over the same period [47].

## Conclusions

With respect to western Kenya, CDC-LT and ITT are the most effective trapping alternatives to HLC for large-scale operational vector sampling. If either method is impractical, due to bulkiness of the trap (ITT) or lack of electricity for recharging batteries (CDC-LT), WET may be an adequate substitute in this context. More broadly, these data suggest that traps actively targeting host-seeking females at night (e g, CDC-LT and ITT) will generally be the most useful for estimating vector biting rates in sub-Saharan Africa, particularly in areas with declining mosquito densities. Novel collection tools such as barrier screens or odour-baited traps, however, may be more appropriate in other regions where malaria is transmitted predominantly outdoors or early in the evening. Identifying which vector sampling methods are effective and practical for large-scale application under different epidemiological settings will require further standardized evaluations of mosquito collection techniques that permit cross-site comparisons.

## Competing interests

The authors declare that they have no competing interests.

## Authors’ contributions

JW performed the statistical analysis and wrote the manuscript. NB and GO assisted in the design of the study and supervised the implementation of the study. GFK participated in the design of the study and helped to draft the manuscript. MJH conceived of the study, participated in its design and coordination, and helped to draft the manuscript. JMV participated in the design of the study and assisted with implementation and supervision of the study. JEG conceived of the study, participated in its design and coordination, advised with analysis, and helped to draft the manuscript. All authors read and approved the final manuscript.

## Supplementary Material

Additional file 1 **Relative rates for capturing female *Culex *spp. stratified by district. **Catch rate (relative to HLC indoor) and 95% confidence interval were calculated using negative binomial regression models. Within each district (column), trap catch rates statistically different from HLC indoor (p < 0.05) are indicated in bold. Across each trap type (row), different letters denote relative capture rates varying significantly (p < 0.05) between districts. Click here for file
