# Supplementary material for: Standardizing operational vector sampling techniques for measuring malaria transmission intensity: evaluation of six mosquito collection methods in western Kenya
Source: Malar J. 2013 Apr 30;12:143. doi: 10.1186/1475-2875-12-143 (PMC3648405; doi:10.1186/1475-2875-12-143)
Supplement: Additional file 1 — Relative rates for capturing female Culex spp. stratified by district. Catch rate (relative to HLC indoor) and 95% confidence interval were calculated using negative binomial regression models. Within each district (column), trap catch rates statistically different from HLC indoor (p < 0.05) are indicated in bold. Across each trap type (row), different letters denote relative capture rates varying significantly (p < 0.05) between districts. [file 1475-2875-12-143-S1.docx]

**Additional file 1. Relative rates for capturing female *Culex* spp.** **stratified by district.** Catch rate (relative to HLC indoor) and 95% confidence interval were calculated using negative binomial regression models. Within each district (column), trap catch rates statistically different from HLC indoor (p<0.05) are indicated in bold. Across each trap type (row), different letters denote relative capture rates varying significantly (p<0.05) between districts.

| Trapping method | Rarieda | Kisumu West | Nyando | Rachuonyo |
| --- | --- | --- | --- | --- |
| HLC indoor | 1.00^*^ | 1.00^*^ | 1.00^*^ | 1.00^*^ |
| HLC outdoor | 0.88 (0.61, 1.28)^a^ | **1.76 (1.19, 2.60)**^b^ | 1.08 (0.94, 1.24)^a^ | **2.95 (2.64, 3.29)**^c^ |
| CDC light trap | **0.16 (0.04, 0.76)**^a^ | **0.24 (0.16, 0.37)**^a^ | **0.22 (0.20, 0.24)**^a^ | **0.24 (0.14, 0.42)**^a^ |
| Ifakara Tent Trap | 0.76 (0.35, 1.66)^a^ | 0.28 (0.07, 1.09)^a,b^ | **0.11 (0.08, 0.16)**^b^ | **0.34 (0.20, 0.57)**^a^ |
| Window exit trap | **0.05 (0.01, 0.43)**^a,b^ | **0.06 (0.04, 0.10)**^a^ | **0.04 (0.03, 0.05)**^a^ | **0.16 (0.08, 0.31)**^b^ |
| Pot resting trap indoor | 0.00^#^ | 0.00^#^ | **0.01 (0.01, 0.01)**^a^ | **0.02 (0.00, 0.16)**^a^ |
| Pot resting trap outdoor | 0.00^#^ | **0.02 (0.01, 0.09)**^a,b^ | **0.01 (0.01, 0.02)**^a^ | **0.07 (0.04, 0.11)**^b^ |
| Box resting trap indoor | 0.00^#^ | **0.01 (0.00, 0.07)**^a^ | **0.01 (0.00, 0.01)**^a^ | 0.00^#^ |
| Box resting trap outdoor | 0.00^#^ | 0.00^#^ | **0.02 (0.01, 0.03)**^a^ | **0.01 (0.00, 0.02)**^a^ |

^*^ Reference collection method within each district

^#^ Trap types that captured zero females were excluded from the model for that district
